# Total Laparoscopic Colopexy for the Treatment of Recurrent Rectal Prolapses in Three Cats

**DOI:** 10.3390/vetsci11080355

**Published:** 2024-08-06

**Authors:** Marta Guadalupi, Claudia Piemontese, Marzia Stabile, Rosanna Dizonno, Francesco Staffieri, Luca Lacitignola

**Affiliations:** 1 Section of Veterinary Clinics, Department of Precision and Regenerative Medicine and Ionian Area (DiMePre-J), University of Bari, 70121 Bari, Italy; marta.guadalupi@uniba.it (M.G.); claudia.piemontese@uniba.it (C.P.); marzia.stabile@uniba.it (M.S.); francesco.staffieri@uniba.it (F.S.); 2 PhD Course Campus of Veterinary Medicine, *Scienze Cliniche Internistiche, Chirurgiche ed Ostetriche Veterinarie* (SCICOV), University of Bari, 70019 Valenzano, Italy; rosanna.dizonno@uniba.it

**Keywords:** laparoscopy, recurrent rectal prolapse, colopexy, cat

## Abstract

**Simple Summary:**

Three cats with recurrent rectal prolapses were successfully treated using total laparoscopic colopexy (TLC). Minimally invasive procedures are increasingly popular in veterinary medicine because of their low postoperative morbidity and quicker recovery. The TLC technique, inspired by laparoscopic-assisted colopexy, involves strategically placed portals to minimize wound complications and ensure effective adhesion of the colon to the abdominal wall. In these cases, non-incisional colopexy using thermal injury was employed to enhance fibrous adhesion, reducing bleeding and avoiding luminal penetration risks. Barbed sutures, used in a continuous single row, facilitated the procedure by eliminating the need for intracorporeal knots, thus reducing surgical time to 30 min. No complications or recurrences were noted during follow-ups. In one case, a viable colopexy was confirmed during a subsequent laparoscopic procedure, demonstrating the technique’s success. Overall, TLC was found to be a feasible, safe, and effective method for treating recurrent rectal prolapses in cats, although further studies with larger sample sizes are necessary to validate these findings.

**Abstract:**

The use of minimally invasive methods has grown in popularity due to decreased postoperative morbidity and a quicker recovery. Colopexy is a surgical method that includes the permanent adhesion of the colonic seromuscular layer to the abdominal wall to avoid rectal prolapses in cats and dogs with viable prolapsed tissues. In this case series, we describe the treatment of three cats with total laparoscopic colopexy (TLC) for recurrent rectal prolapses. A non-incisional colopexy was created by suturing the colon to the abdominal wall with a barbed suture. There were no intraoperative complications and a 6-month follow-up revealed no prolapse recurrence. Our study demonstrates that TLC approaches are feasible, safe, and free of problems when used to treat recurrent rectal prolapses in cats, although a larger caseload is required to validate the results obtained from our reported cases.

## 1. Introduction

Colopexy is a surgical technique that consists of the iatrogenic creation of a permanent adhesion of the colonic seromuscular layer to the abdominal wall [1,2].

This procedure has been indicated in the prevention of the recurrence of rectal prolapses in dogs and cats with viable prolapsed tissues [2]. Rectal prolapse is a common occurrence in animals that are experiencing severe diarrhea and infestation [2]. Additionally, persistent tenesmus may also result in rectal prolapse caused by several intestinal, uterine, and urinary bladder conditions [3,4]. Furthermore, rectal prolapse may also occur after perineal hernias are repaired, particularly in dogs whose bilateral hernias are compounded by rectal sacculation. Colopexy is also one step of three [5] in the treatment of perineal hernias in dogs [1].

An open surgical procedure has been successfully described in dogs and cats [2]; furthermore, a silicone elastomer sling was also suggested as a possible alternative to other open techniques [6]. With the introduction of new minimally invasive techniques, laparoscopic-assisted colopexy (LAC) [7,8,9] and total laparoscopic colopexy (TLC) [10,11] surgeries have been used on small animals. To the best of our knowledge, total laparoscopic colopexy has only been reported for dog species. For this reason, we aimed to describe the surgical techniques and outcomes of three cases of recurrent rectal prolapses in cats treated with total laparoscopic colopexy.

## 2. Materials and Methods

### 2.1. Case 1

A ten-month-old female European cat weighing 3.2 kg was admitted to the Veterinary Teaching Hospital (VTH) of the University of Bari for red soft tissue exiting the anus 3–4 weeks prior to admission. The owner confirmed that the cat had maintained its appetite, its feces were smooth and regular, and anthelminthic prophylaxis was inconsistent. A clinical evaluation revealed the presence of a rectal prolapse with viable tissue that was easily reduced using a digital maneuver. The hematological and serum biochemistry results revealed no abnormal findings. As a primary treatment, a purse-string suture was administered under sedation after the digital prolapse reduction. The suture was removed 48 h after hospitalization and the prolapse recurred. Thus, in accordance with the permission of the owner, TLC was planned.

### 2.2. Case 2

A one-year-old European male cat weighing 3 kg was admitted to the VTH for a recurrent prolapse that had previously been unsuccessfully treated with a manual reduction two months before presentation and had recurred after a purse-string suture failed. During the clinical examination, the rectal mucosa prolapsed 2–3 cm from the anus. The tissue was viable and could easily be reduced using a digital maneuver. Due to the prolapse recurrence, the owner was advised that TLC should be performed on the cat. Pre-surgical blood and serum analysis were performed. Surgery was scheduled 48 h after the evaluation. 

### 2.3. Case 3

A three-year-old European female feral cat was admitted to VTH because of recurrent rectal mucosa exiting from the anus as a consequence of a previous surgically treated perineal hernia three weeks before admission. The cat was clinically evaluated, and X-rays confirmed small rectal deviation and constipation was confirmed. A pursue-string suture was applied for 48 h, but recurrence of rectal mucosa prolapse was observed. In accordance with the cat’s care giver, a TLC was planned. Blood and serum analyses were carried out prior to surgery.

### 2.4. Surgical Procedure

Similar surgical procedures were performed in all cases. The cats were treated with lactulose for two days before the planned surgery and fasted for 24 h before the surgical procedure. Under sedation, the prolapse was revaluated for tissue viability and manually reduced. Before the surgery, a complete physical examination, hematology, and biochemistry checks were within the normal range and an American Society of Anesthesiologists’ status II was assigned. The cats were premedicated with dexmedetomidine (0.005 mg/kg; Dexdomitor 0.5 mg/mL; Orion Srl, Veggiano, Italy), buprenorphine (0.01 mg/kg; Bupredine 0.3 mg/mL; Dechra, Milan, Italy), and alfaxalone (2 mg/kg; Alfaxan multidose 10 mg/mL; Dechra, Milan, Italy); these were administered intramuscularly. Approximately 20 mins later, the cephalic vein was catheterized with a 22 G intravenous catheter. Cephazoline (8 mg/kg; IV; TEVA, Milan, Italy) was administered as a prophylactic antibiotic therapy. Anesthesia was induced with alfaxalone to the required effect. A total volume of 0.1 mL lidocaine (2% lidocaine; Ecuphar Italia Srl, Milan, Italy) was administered to the arytenoid and the cats were intubated with a cuffed endotracheal tube (4 mm ID). Once the patients had been connected to a non-rebreathing circuit, anesthesia was maintained with isoflurane (Isoflo; Zoetis Srl, Milan, Italy) administered with 100% oxygen. Monitoring was performed using a multiparametric monitor (C80-V Veterinary Multiparameter Monitor Comen; Foschi Srl, Bologna, Italy), which included electrocardiography, capnography, inspired and expired concentrations of isoflurane, non-invasive blood pressure, pulse oximetry, and esophageal temperature. Each parameter was recorded every five minutes during surgery. Lactated Ringer’s solution (Ringer Lattato; Fresenius Kabi Italia Srl, Milan, Italy) was administered at a constant rate of 3 mL/kg/h IV throughout surgery. Surgery was continued after approximately 5 min.

**Figure 1:**
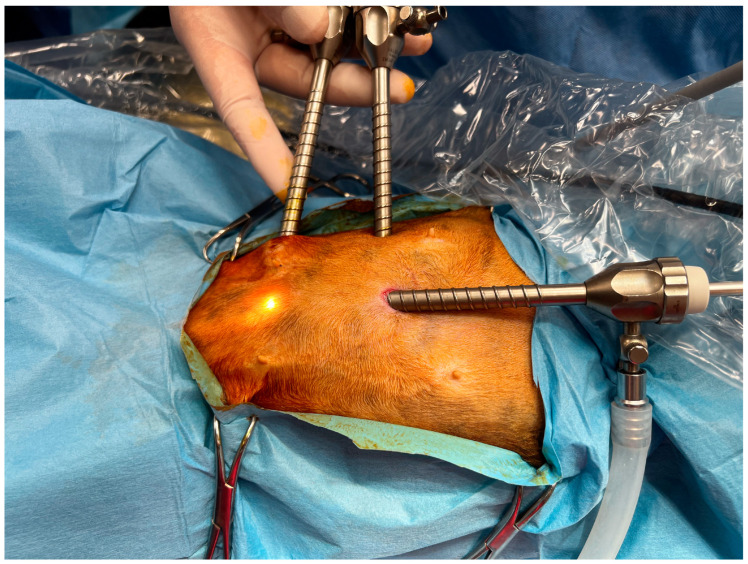
Port Placement. Cranial on the right of the picture. Optical port at the umbelical scar. Operative ports were placed on the right side. The cranial operative ports at the level of first port 3–4 cm to the right. The caudal operative port was placed three cm caudally to the second cannula.

The abdomen was routinely aseptically prepared from the xiphoid apex to the pubis. The cats were positioned in dorsal recumbency. A laparoscopic cannula (KarlStorz, Tuttlinghen, DE, Germany) with a width of 5 mm and blunt trocar were inserted 5 mm distal to the umbilical scar using a modified Hasson technique. Once proper cannula placement was confirmed, pneumoperitoneum was established by CO_2_ flux at 1.5 L/min and the maximum intrabdominal pressure was set at 4 mmHg. A 30° telescope (KarlStorz, Tuttlinghen, DE, Germany) that was 5 mm wide was inserted into the cannula and a visual inspection of the abdomen was performed. No entry-related injuries to the abdominal organs were observed. Under a visual inspection, two other 5 mm cannulas were inserted into the abdominal wall. A cannula was placed at the level of the optical portal, but placed 3–4 cm to the right. The third cannula was caudally placed 3–4 cm from the second one (Figure 1).

**Figure 2:**
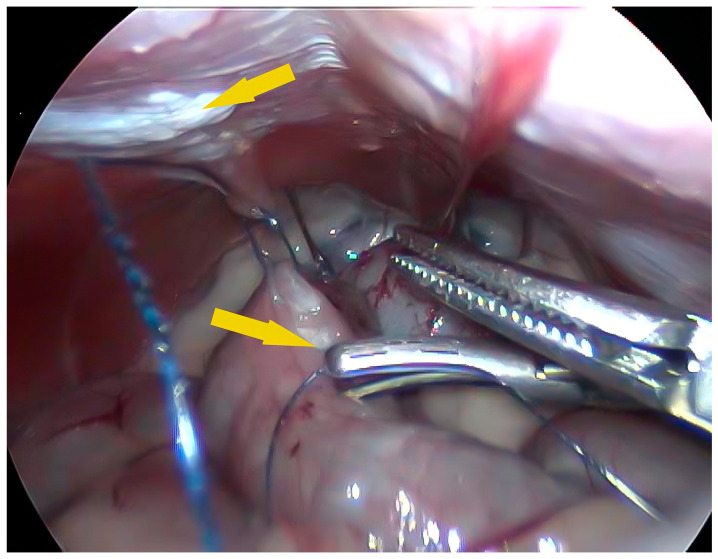
Intraoperative image, during scarification of colonic serosa and left transversus abdominis. Yellow arrows show the serosa appearance after HS activation.

Two grasping forceps were inserted into the second and third cannulas. The distal colon was inspected for any serosal damage or vascular supply abnormalities. The distal colon was then cranially retrieved 2–3 cm to the left of the hemiabdomen to locate a proper area for the final position of the colon to be fixed on the abdominal wall. Subsequently, a 3-0 USP PDS barbed suture (V-loc 180; Medtronic, Milan, Italy) was inserted through the caudal cannula using a laparoscopic needle driver. A first suture was placed, which passed into the transversus abdominis aponeurosis at the previously evaluated location and through the corresponding colonic area on the antimesenteric border. Careful attention was paid to include only the seromuscular layer and to avoid passing into the colonic mucosa. The needle was passed into the weld loop tail of the suture, and the tissues were gently moved to avoid traction. After completely passing through the loop, the suture wire was pulled, and the colon was gently affixed to the abdominal wall. At this point, the needle driver was removed and a harmonic scalpel (HS; Ultracision, J&J, Cincinnati, OH, USA) was inserted through the same portal. The abdominal wall serosa and corresponding colonic area were scarified by several activation spots of the HS along an ideal line of 3–4 cm (Figure 2).

**Figure 3:**
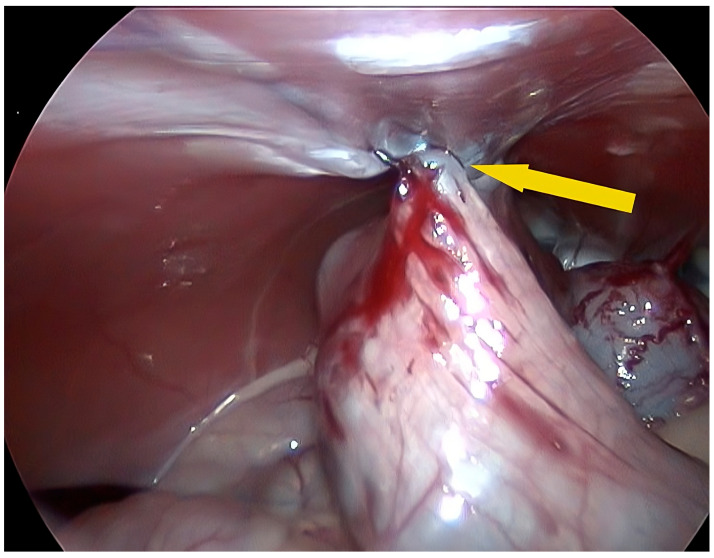
Intraoperative image of the completed colopexy (yellow arrow).

The HS handpiece was retrieved, and the needle driver was reinserted to complete the suture. Five loops were completed in a caudocranial direction along the previous scarification line. Ideal tension was applied to the suture wire to allow the correct colonic apposition to the abdominal wall. A final suture was administered in the opposite direction to avoid the possibility of losing adequate tension on the wire or a barb failure. Once the colopexy was completed, a laparoscopic scissor was introduced through the caudal cannula instead of the needle driver and the wire was cut 7–8 mm from the final loop. The needle was removed from the abdomen through the cannula. The correct position of the colopexy and adequate tension were evaluated by gentle manual traction on the colon (Figure 3).

The pneumoperitoneum was then interrupted, and CO_2_ was removed by gentle pressure on the abdominal wall. The cannulas were then removed. The portal incisions were sutured with 3-0 USP PDS using double-layer, single-interrupted stiches.

The entire procedure was completed in 30 min (skin to skin).

## 3. Results

The cats were hospitalized for four days following surgery. The post-operative regimen included four days of robenacoxib at 1 mg/kg (Onsior; Elanco Italia s.p.a, Milan, Italy) as an analgesic therapy. Lactulose was administered for a week BID per OS and a low-fiber diet was suggested. The feces were normal and there was no recurrence during the hospitalization. The skin sutures were removed at the first follow-up, 14 days after surgery. There was no indication of a rectal prolapse upon clinical examination.

**Figure 4:**
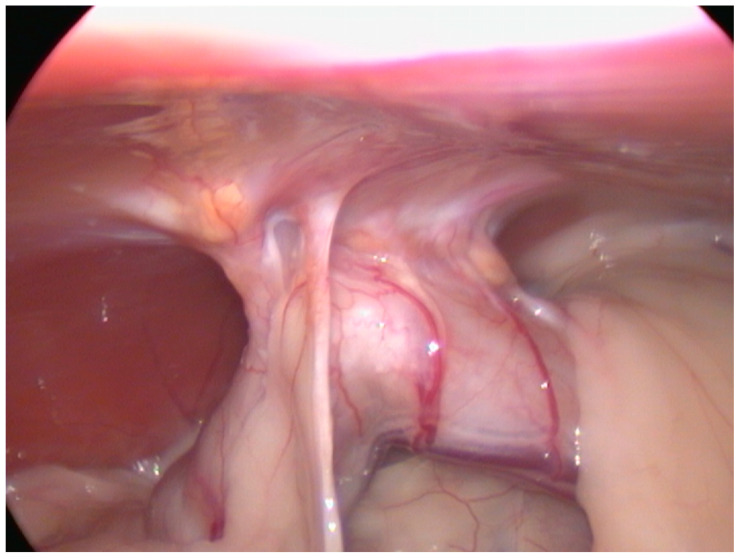
Colopexy appearance 4 weeks after TLC, in Case 3. Complete adhesion was visible between colon and abdominal wall.

In Case 3 the cat was laparoscopically spayed 4 weeks after TLC. The colopexy was thus rechecked and it was stable and in place, and a permanent adhesion of colon to the abdominal wall was appreciated; the serosa covered the pexy and vascularization was clearly visible. No evidence of suture material was seen at this time (Figure 4).

There was no recurrence nor any abnormalities at the 6-month post-operative examination all cases.

## 4. Discussion

The cases reported relate to three cats treated with TLC for recurrent rectal prolapses. Colopexy is the preferred treatment for cats with rectal prolapses due to the high prevalence of the development of postoperative strictures after rectal amputation [1] in the case of viable tissues and allowed reductions [2]. Colopexy may be required in patients with rectal prolapses who have been previously treated with a purse-string suture that has previously failed or that cannot be manually corrected [1,2,3,4], as observed in the current cases.

Minimally invasive procedures (MISs) are becoming widely available for veterinary patients [12]. Many surgeons have adopted the use of MIS techniques due to lower postoperative morbidity and a faster recovery [13]. LAC was demonstrated to be feasible and resulted in a sero-serosal adhesion between the colon and abdominal wall 11 weeks post-procedure in dogs in experimental studies [7,11]. The main complications observed were related to wound dehiscence or the recurrence of rectal prolapses due to colopexy failure [14]. Recently, Park et al. [10] reported the case of a 2.7 kg Maltese dog treated with total laparoscopic colopexy for a recurrent rectal prolapse.

The main difference between LAC and TLC is the placing of portals on the opposite site. In the LAC procedure, the caudal portal used to grasp the colon is located on the left hemiabdomen. The portal may be enlarged from 2 cm [9,14] to 8 cm [11] to permit correct suture application and to create stable adhesion. In our cases, we adopted a similar portal setting to the case described by Park et al. [10]. This positioning of the portals allows the colopexy suture line to be located away from the suture of the abdominal fascia and skin. This decreases the possibility of wound dehiscence or skin wound complications that may result in colopexy failure. The position of the instrumental portal permits adequate maneuvers and no inter-reference with the telescope during the suture pattern construction.

In our cases, a non-incisional colopexy was created by HS application. No significant differences have been described between incisional and non-incisional colopexy procedures [2,7]. The use of a thermal injury to enhance the fibrous adhesion reduces bleeding, eliminates the risk of inadvertent luminal penetration during cutting, and provides a reference point whilst suturing the stomach [15,16,17,18] and colon [7] to the body wall.

The type of suture pattern used to ensure that the colon adhered to the body wall during healing was a continuous pattern [9]. The suture was positioned in single or double rows. In laparoscopic gastropexy, both single and double rows demonstrated similar results [18]. In the current cases, we used a single row of barbed sutures to affix the colon to the abdominal wall. The benefits of barbed sutures include a lack of the need to perform intracorporeal knot tasks; thus, the procedure is less technically demanding and lowers the surgical time [15,18,19]. Although surgery was completed without conversion to open surgery in a reasonable time with the use of a knotless suture material, the procedure required surgeon dexterity due to a reduced working space. In the cases we treated, the time to complete the surgery was 30 min (skin to skin); other authors have reported longer surgery, with a mean average of 40 to 60 min [7,9,11].

No surgical site complications were observed in either case, and no recurrence was observed at the available follow-ups.

In Case 3, a laparoscopic re-examination of the pexy was possible because the cat was neutered 4 weeks after the TLC. At this time, a viable, firm and stable colopexy was observed, confirming that the TLC technique had been successfully performed to create a permanent adhesion while the healing time of the colopexy was comparable to other pexy techniques [17].

Additionally, the cats treated in Cases 1 and 2 were young animals with an inconsistent history of anthelmintic prophylaxis. For these reasons, appropriate anthelmintic therapy should be combined with surgical treatment to reduce the recurrence rate and improve surgery outcome [20]. Tenesmus can induce rectal prolapse owing to a variety of disorders, in addition to intestinal parasitosis, including neoplasia and foreign bodies, dystocia, urolithiasis, constipation, congenital defects, prostate disease, and rectal polyps [1,2,3,4,21].

As a result, surgery should be incorporated in a comprehensive treatment plan focused at addressing the underlying disease causing the prolapse.

## 5. Conclusions

The TLC techniques were feasible, safe, and free of complications when treating recurrent rectal prolapses in cats. A larger caseload is required to confirm the results obtained in this short case series.

## Data Availability

All data were included in the manuscript.
